# Developing a novel co‐produced methodology to understand ‘real‐world’ help‐seeking in online peer–peer communities by young people experiencing emotional abuse and neglect

**DOI:** 10.1111/hex.13621

**Published:** 2022-10-10

**Authors:** Vanessa Bennett, Chloe Gill, Pam Miller, Peter Lewis, NeurOX YPAG, Catherine Hamilton‐Giachritsis, Iris Lavi

**Affiliations:** ^1^ Neuroscience, Ethics and Society Group, Department of Psychiatry University of Oxford Oxford UK; ^2^ Research and Evidence Team NSPCC London UK; ^3^ Department of Psychiatry University of Oxford Oxford UK; ^4^ Department of Psychology University of Bath Bath UK

**Keywords:** children and young people, co‐production, emotional abuse, help‐seeking, neglect, online, peer support

## Abstract

**Background:**

Recent systematic reviews suggest mediating factors (barriers and facilitators) of help‐seeking for children and young people (CYP) with a range of mental health problems but highlight the need for a more robust methodology underpinned by theoretical frameworks. Emotional abuse and neglect is the most prevalent and pervasive form of abuse, with many CYP remaining unknown to professional services. These CYP are underrepresented in help‐seeking research yet seek help from their peers via anonymous online publicly available message communities.

**Objectives:**

To sensitively co‐develop qualitative methodology to explore ‘real‐world’ data to inform our understanding of help‐seeking for these CYP, and define potential mediators (barriers and facilitators) and mechanisms of change associated with online peer support.

**Methods:**

Co‐production with 10 young co‐researchers (YCoR; aged 14–18 years) from the NeurOX Young People's Advisory Group (YPAG) included co‐development and triangulation to apply different research methods (i.e., interpretative phenomenological, thematic and conversation analyses) to analyse rich ethnographic material from 20 publicly available online message conversations between help‐seekers experiencing or questioning emotional abuse and neglect. A theoretical model of adolescent help‐seeking proposed by Rickwood et al. was used as a conceptual framework to guide methodological development.

**Results:**

The methodological approach facilitated the identification of barriers and facilitators of help‐seeking contextualized to the lives of these CYP: understanding and validating of abuse, emotional competence, fears and uncertainties around disclosure, knowledge, motivational factors and connection/trusted relationships. Notably, positive changes in expressed or perceived ‘psychological state’ and/or intention to seek help were noticed in 9 of 10 message threads that included a ‘conversation’ (≥3 posts). Themes associated with change related to connection with supportive peers; compassionate responding and the safeness of the online community. The existing adolescent help‐seeking model was found to be too simplistic to account for help‐seeking associated with emotional abuse and neglect.

**Conclusion:**

The novel methodological approach offers a meaningful way to explore ‘real‐world’ data with YCoR, for a population underrepresented in help‐seeking research. Proposed relational mechanisms involve connection, compassion and online communities. Further research coproduced with YCoR with diverse care experiences and characteristics is required to upscale the methodology and further validate and extend the findings.

**Public Contribution:**

The core study was co‐produced with 10 YCoRs from the NeurOX YPAG who have been involved in over 135 h on and offline work. Their roles involved co‐deciding the direction of the study, evolving methodology, detailed co‐analysis and reflective processes throughout all aspects of the study, interpretation, presentation and discussion of the findings with the NSPCC and Childline, and involvement in all communications. Additional consultation and involvement included further interested members of the NeurOX YPAG for the final online workshop and dissemination outputs.

## 1 INTRODUCTION

Emotional abuse and neglect, including witnessing or experiencing domestic abuse, is the most pervasive, prevalent and yet under‐reported form of harm affecting children and young people (CYP) from any background.^1^, ^2^ Left unaddressed they can be associated with short‐term distress and greater risks for long‐term mental and physical health sequelae.^3^, ^4^ However, seeking help and engaging with support services for these CYP is complex.^5^

### 1.1 Research methodology exploring help‐seeking for CYP

Help‐seeking research for CYP who are struggling psychosocially is an emerging field of research. Most studies have focussed on intentions to seek help in adolescent school‐based populations. In a systematic review of help‐seeking for common mental health problems, only 7 of 54 studies included adolescents who had accessed services.^6^ A number of recent systematic reviews have suggested mediators (barriers and facilitators) of help‐seeking, by adolescents for a range of mental health problems, including an individual's capabilities and motivational factors, social aspects, relational factors and systemic or structural factors including availability and opportunity to receive support.^6^, ^7^, ^8^ However, what is missing from the evidence base is the robust methodology and a clear understanding of the mechanisms that drive help‐seeking for CYP, how these are moderated at the individual, social and environmental levels and how these components may interact to affect help‐seeking behaviour on‐ and offline for CYP.^5^, ^6^, ^9^, ^10^ What is known is that some CYP with diagnosable and highly stigmatized mental health conditions are often unaware of, or are the most reluctant or afraid to discuss, their problem or needs.^10^, ^11^, ^12^

### 1.2 Help‐seeking for CYP experiencing emotional abuse and neglect

For many CYP experiencing emotional abuse and neglect, maladaptive safety strategies associated with trauma, internalization of emotions and their unsupportive and uncaring environments may have a significant influence on help‐seeking.^5^, ^13^ Maladaptive strategies may also contribute to the unfathomable, or often unspeakable, psychosocial world for some CYP and hence may influence their capacity to engage and build trusting relationships necessary to facilitate disclosure and progress to find and maintain support.^5^ Consequently, evidence, in their own uninhibited language, from CYP most in need of support describing their experiences of help‐seeking, would be valuable to inform multidisciplinary research.^5^, ^14^, ^15^, ^16^ Such knowledge and understanding of how CYP may formulate intentions and motivations to seek help would improve our understanding of their needs and enable better tailoring of formal and informal (i.e., nonprofessional) support services.^5^, ^9^ However, such evidence is often limited by practical, ethical and methodological challenges outlined in the literature.^16^, ^17^, ^18^, ^19^

### 1.3 Role of online help‐seeking and peer support

Some studies suggest that online peer–peer services resonate better with CYP's lifestyles and preference to connect with similar others, receive some comfort for their distress, and help validate their problems.^20^, ^21^ Although many CYP experiencing emotional abuse and neglect are likely to remain unknown to professional services,^22^ some appear to engage with informal online support services.^18^ Such engagement suggests that these online services may offer many CYP experiencing abuse an opportunity to explore and engage with these supportive communities. These informal, anonymous and confidential environments may facilitate help‐seeking and disclosure by removing some barriers associated with environmental and psychosocial fears.^8^, ^20^, ^23^ These CYP may confide in their peers, rather than authorities and adults.^24^ Online services provide a potential opportunity to offer immediate support and develop CYP's agency and capabilities to seek further professional help.^20^ Such online environments, when perceived to be safe to explore, supportive and trustworthy, have been shown to facilitate beneficial peer–peer emotional support for isolated CYP with highly stigmatized mental health problems (i.e., suicide and self‐harm) through connection to others within those online communities.^21^, ^25^ Despite this, research exploring online peer–peer forums is limited; and reported to lack appropriate measures of help‐seeking.^26^, ^27^ A recent report, including grey literature, identified several gaps in research for non‐face‐to‐face help‐seeking for CYP.^10^ To the author's knowledge, detailed methodology and research on help‐seeking and the role of peer support via online message forums for CYP experiencing emotional abuse and neglect have not been reported internationally.

### 1.4 Methodology to explore online help‐seeking

While some message boards have been set up specifically for a research study,^25^ few studies have explored data from active publicly available message board communities offering informal peer support services.^21^ Therefore, reliable methods for exploring these ‘real‐world’ (i.e., not designed for research purposes) data sources have not been reported. Qualitative methods, such as interpretative phenomenological analysis^18^ and reflexive thematic analysis of message posts^28^ may facilitate the generation of knowledge about the context and experiential qualities relating to an individual, writing at that moment. However, interaction in online forums to discuss personal problems add a further layer of complexity in terms of environment (community and functionality), language, time and space. Given that conversations form the basis of social and therapeutic interactions, some researchers have begun to apply conversation analysis to look at interactions in online forums.^29^ The following aspects appear most relevant to explore in developing a methodology to look at online message boards:

(1) sequence organization of thread—turn‐taking, and who talks to who and when^25^, ^30^;
(2) composition of thread/interactions and coherence—presenting problems, questions, flow and sense in sequential talk^25^, ^31^;
(3) timings of interactions and role of technology^25^;
(4) relationship building and rapport—emotive qualities of the conversation^25^, ^30^;
(5) advice‐giving in the conversation.^25^, ^31^, ^32^

### 1.5 Theoretical frameworks to guide help‐seeking methodology

Recent literature has highlighted the absence of theoretical help‐seeking frameworks to guide the design of online help‐seeking interventions for adolescent mental health problems and adverse childhood experiences.^5^, ^6^, ^20^ However, Rickwood et al.^11^ defined a process for help‐seeking for adolescents suggesting that this highly personal goal involves a series of social transactions that interface between an individual's thoughts and feelings with those of another in a social relationship. Thus, help‐seeking is explained as a ‘process whereby the personal becomes increasingly interpersonal’ (p. 8).^11^ This has also been considered in online help‐seeking by Pretoruis et al.^20^ They proposed that this could be combined with self‐determination theory^33^ and outlined clusters of benefits and limitations of online help‐seeking that drive motivation and well‐being for adolescents. These are related to the fulfilment of primary needs of self‐determination: autonomy, competence and relatedness.^20^ It may be that CYP need to obtain a degree of competency across a series of stages to progress and actively seek out professional support.^11^, ^20^

### 1.6 Research aims and objectives

The overarching aim was to develop co‐produced methodologies to analyse these ‘real‐world’ data to enhance our understanding of the psychosocial characteristics and help‐seeking journeys of CYP experiencing emotional abuse and neglect who engage with their peers in an online message board service. To provide a theoretical framework, the process defined by Rickwood et al.^11^ and elaborated by Pretorius et al.^20^ was utilized to explore the online help‐seeking journey or ‘process’.

This paper is one of three reporting on a study co‐produced with young co‐researchers (YCoR). The aims and research questions addressed across the three papers are shown in Table 1. The current paper (Paper 2) describes the co‐development and application of the qualitative methodology to explore mediators to help‐seeking (barriers and facilitators) and the role of peer–peer interactions.

**Table 1:** Aims, research questions and methodologies for different aspects of the research study reported across three papers

Paper	Study and aims	Research questions	Methodology applied/developed
Paper 1^18^	To understand the context and experiences of children and young people seeking help relating to their experiences of emotional abuse and neglect via an anonymous, online peer–peer message forum	What are the contexts, language, mental health challenges, explicit and inferred help‐seeking motivations of children and young people seeking help relating to their experiences of emotional abuse and neglect?	Familiarization, data sampling strategy and data selection from publicly available ‘real‐world’ data sources: working with a Young People's Advisory group
			Co‐produced phenomenological interpretative analysis of online message board discourse (development of a reflexive group co‐analysed process)
Paper 2 (current paper)	To understand the help‐seeking journeys, and the role of peer support for children and young people engaging with an online peer–peer message board	What evidence is available for change following interactions? How can positive or negative changes be defined?	Reflexive thematic analysis of contextualized content of message posts
			Conversation analysis of ‘chat’ features and elements of peer interactions via online message boards
		What mediators (barriers and enablers) of help‐seeking can be inferred from message board posts/threads for CYP who describe experiences of emotional abuse and neglect?	
			Application of theoretical framework to methodological development to guide help‐seeking research
		What qualities of interactions/conversations moderate the help‐seeking journeys for CYP seeking help for these experiences from their peers?	
		How do mediators relate to the help‐seeking model?	
		What are potential mechanisms of peer support?	
Paper 3^34^	Case study illustrating the involvement approach, adapting research methodology, building capabilities and evaluating involvement and impact	What is the meaningful role young co‐researchers in co‐production?	Participatory/co‐production methodology
			Evaluating different perspectives on young co‐researcher's involvement and defining impacts
		By addressing:	
		Flexible involvement methodology to build capabilities	
		Adapting research methodology to embed lived experience throughout the project	
		How to evaluate involvement and impact	

## 2 METHODS

### 2.1 Funding and ethics

The research reported in this paper was developed as part of a 3‐month cross‐sector research placement with the NSPCC funded by the UKRI Emerging Minds network and the Department of Psychiatry/University of Oxford. The study involved retrospective analysis of anonymous (pseudonymized) online publicly available ‘data’ from the Childline online peer‐support message boards. The study was approved by NSPCC Research Ethics Committee (R‐20‐189, 2020) with reciprocal approval by the University of Oxford Central University Research Ethics Committee (Ref:R62044/RE001). Confidentiality for members is maintained. Only pseudonyms were known to researchers; these and any names mentioned in messages were changed in all internal and external reporting (further information is published in Bennett et al.^18^). Direct text from potentially active young service users is not shared to protect their identity, privacy and safeguarding in public reporting of this sensitive research. These data have been through an external blind peer review process conducted by the NSPCC in a full internal NSPCC report.

### 2.2 Involvement of YCoR in the co‐design of methodology

Full details of the YCoR recruitment, characteristics and co‐production involvement methodology are detailed elsewhere.^34^ In summary, 10 YCoR from the NeurOX Young people's Advisory Group (YPAG; in the Neuroscience, Ethics and Society Group in the Department of Psychiatry and University of Oxford), some of whom had used the service, were enlisted to the research project team and followed the group's agreed principles of co‐production and terms of reference.^35^ NVivo (v12) qualitative software was used by one of the adult researchers (V. B.) to annotate and code extracts of the text and organize identified themes as they were discovered; data were exported to Excel to share with YCoR. Zoom, Padlets (https://en-gb.padlet.com), Google sheets and Google docs were used with YCoR for online and offline working.

### 2.3 The message board ‘data’

The ‘data’ comprise of series of messages initiated by an anonymous (pseudonymized) help‐seeker on the Childline message boards. Help‐seekers may receive online responses from a peer supporter(s), occasionally a Childline moderator, or another help‐seeker—forming a ‘message thread’. Moderators (Childline staff) were rarely involved in threads and analysis was considered beyond the scope (resource and time available) for this first exploratory project.

Terminology to describe emotional abuse and neglect from research literature^13^, ^36^ and online sources (NSPCC) were utilized to inform message thread selection. (Further details and definitions of abuse are included in Supporting Information: Data Table 1.) Inclusion criteria, data selection, sampling and the role of YCoR in the process are described in detail in the companion papers.^18^, ^34^

### 2.4 Study design, data handling and methodological development

**Figure 1:**
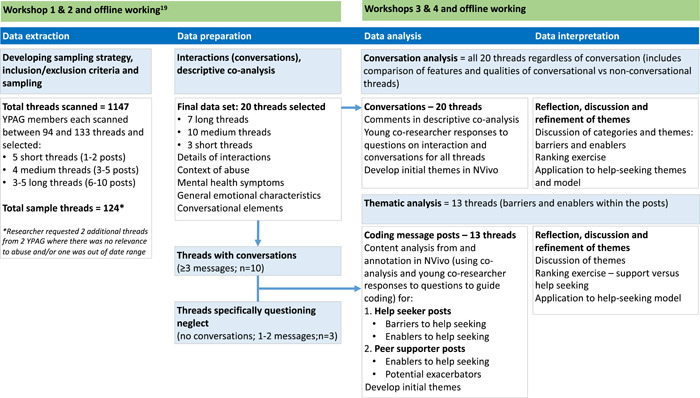
Overview of study design and data handling. *Researcher requested 2 additional threads from 2 YCo where there was no relevance to abuse and/or one was out of date range. *Source*: Adapted from Bennett et al.^18^

An overview of the study design, data handling and different qualitative methodologies are summarized in Figure 1. The first step in this process was to ‘describe’ the data before developing the qualitative methodology.

#### 2.4.1 Characterizing peer–peer interactions and co‐developing research methods

Initially, the data set was characterized according to types, numbers and patterns of help‐seeker, peer and moderator interactions, as well as response timing for all included message threads (*n* = 20). This step was important to decide: (i) how thematic and conversation analytic methods could be applied; and (ii) how ‘change’ could be explored and investigated. ‘An interaction’ was defined as an exchange between a unique help‐seeker–peer supporter or peer supporter–peer supporter pair (i.e., at least two posts). ‘A conversation’ was defined as a series of interactions involving at least three posts (i.e., at least two interactions).

Based on this step, given the short project duration and focus on interactions associated with peer support, it was decided to perform reflexive thematic analysis on threads with conversations (*n* = 10). An additional three threads specifically questioning neglect were included; to explore the content for these CYP and why it may not elicit an interaction. Conversation analysis examined what may have enabled and inhibited interactions so all threads were included. Quantitative analysis of interactions and change was not performed due to limitations of project duration, scope and sample size.

### 2.5 Qualitative research approach and methodologies

The theoretical help‐seeking framework and methodology are described. Applying insights and findings from the familiarization exercises and interpretative phenomenological analysis,^18^, ^37^, ^38^ a methodological approach was developed to scaffold YCoR learning and involvement in thematic and conversation analyses.

#### 2.5.1 Applying the theoretical framework and integrating YCoR interpretations

**Figure 2:**
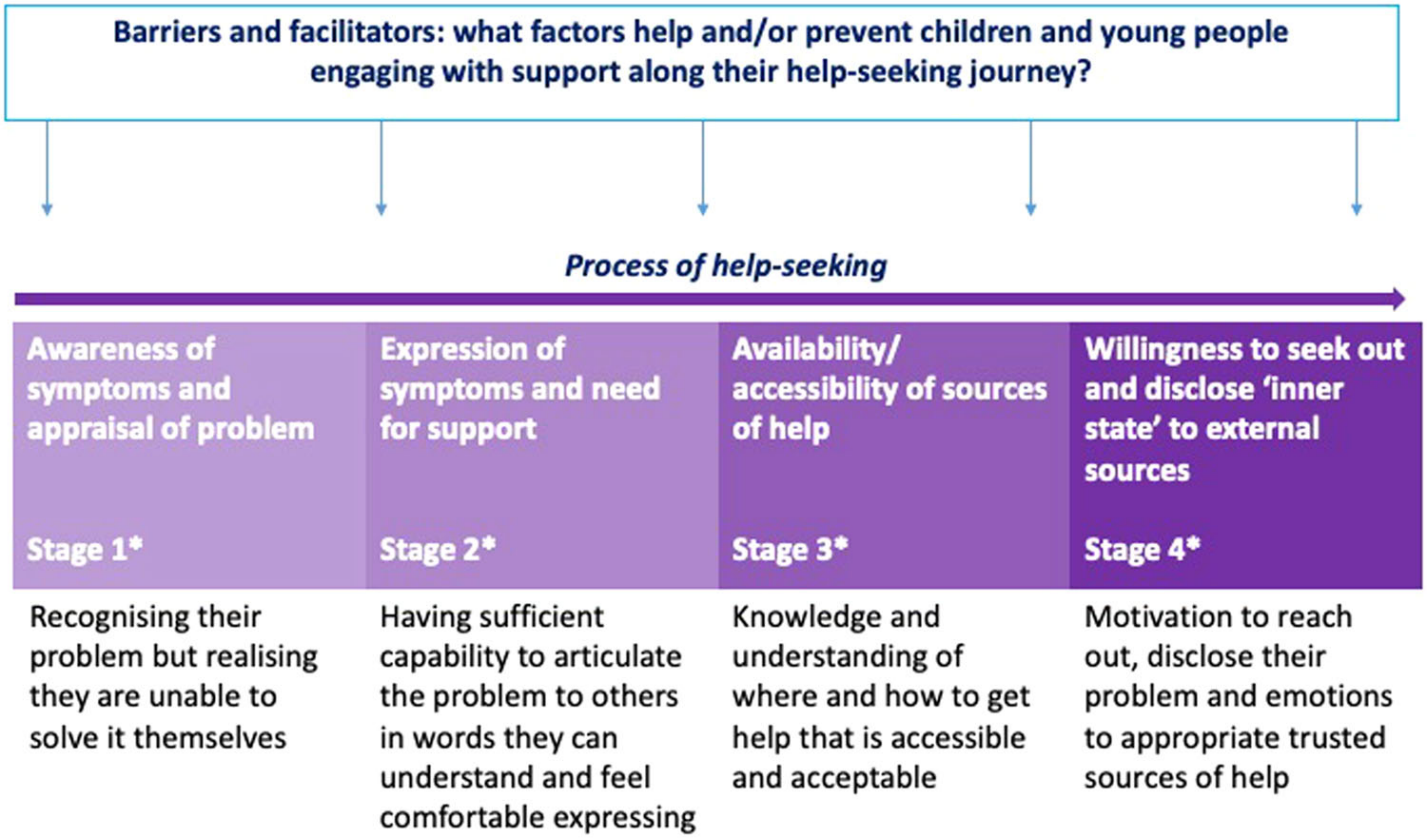
Application of help‐seeking theory to understand the journeys of young people on the Childline message boards. *Stages labelled to facilitate the study methodology and application of the help‐seeking model with young co‐researchers. *Source*: Adapted from Rickwood et al.^11^ and Pretorius et al.^20^

A theoretical framework based on the research by Rickwood et al.^11^ and Pretorius et al.^20^ was shared with YCoR and used to explore mediating and moderating factors for help‐seeking as shown in Figure 2.

To scaffold learning and facilitate reflexive processes, open questions (Box 1) were embedded throughout each thread (after each message post) for the YCoR to reflect on and respond to for (a) the reflexive thematic analysis to identify potential mediators (barriers and enablers) to help‐seeking from help‐seeker and peer supporter posts; (b) the conversation analysis to explore qualities and features of the conversation that were considered to be helpful or unhelpful (by YCoR). The framework and questions provided a structure and prompt towards noticing, evidencing and defining ‘change’ against a process for individuals throughout threads.

**Questions for YP embedded in the threads analysed by the researcher**

**Table:** 

A. Thematic analysis: Help‐seeking questions (relating to Rickwood et al.^11^ help‐seeking process)
For the first help‐seeking post:
(1) Where do you think they are in the help‐seeking process? (2) What key evidence makes you think this? (3) Can you suggest any potential barriers (what may be stopping them) and the facilitators (what may be motivating them) to seeking help at any of these stages?
For peer‐supporter posts:
(1) What do you think may be most helpful to encourage help‐seeking? (2) What do you think may not be helpful?
For subsequent help‐seeking posts:
(1) Where do you think they are in the help‐seeking process now (what do you think has changed)? (2) What key evidence makes you think this—relating to barriers and facilitators?
B. Conversation analysis
(1) How do you think the timings of the interactions may have impacted on the flow of conversation? (2) How do you think these could impact on feeling supported? (3) How could the sequence and flow of conversations impact on help‐seeking (think in terms of what could be a barrier and facilitator)? (4) How is the reply function used in the thread? How does it add clarity to, or confuse, the conversation? (5) What features of the text/response do you think are most important to encourage an interaction/longer conversation? (6) What features of the text/response do you think stops a conversation happening? (7) What happens to the conversation when other ‘posters’ (help‐seekers or peer supporters) ‘jump’ in between a pair having a conversation?

#### 2.5.2 Reflexive thematic analysis

This flexible thematic analytical process^28^ between YCoR and the adult researchers (V. B. and C. G.) was applied to analyse selected message posts (*n* = 13; as per Figure 1); providing a snapshot of the psychosocial characteristics that were thought to be related to these help‐seeking mediators in a specific moment. For this short study, the adult researcher utilized YCoR reflections (from phenomenological analysis reported separately),^18^ and YCoR responses to embedded questions (Table 2) to guide the coding of content and generate themes in NVivo. Themes inferred from the data and a coding structure were developed within the following high‐level categories: (i) help‐seeker posts—barriers; (ii) help‐seeker posts—enablers; (iii) peer supporter posts—barriers; (iv) peer supporter posts—enablers (see detail in Table 3).

**Table 2:** Mediating factors (barriers and facilitators) of help‐seeking inferred from help‐seeker and peer‐supporter messages

Help‐seeker posts—barriers	Help‐seeker posts—facilitators	Peer supporter posts—facilitators^a^	Peer supporter posts— exacerbate barriers
**Unable to understand, self‐validate or accept experience of abuse and/or neglect**	**Understanding and knowledge to enable self‐validation and move towards acceptance of abuse** a	**Offering knowledge and shared experience to help validate and move towards acceptance of abuse/reduce social stigma**	**Lack of understanding or sensitivity to HS experience, invalidating abuse, or inducing social comparison of experience**
**Poor emotional competence**	**Adequate emotional competence**	**Enhance emotional competencies**	**Negatively impact emotional competence**
Unable to describe or express emotions: possible emotional dysregulation	Identify and talk about emotions, mental health symptoms and problems	Encourage talking about/expressing emotions	
Lack of understanding of distressing emotions associated with abuse:	Understanding emotions associated with emotional manipulation and abuse	Using own compassionate skills to soothe negative emotions and distress:	Exacerbate distress through lack of compassionate skills and responding:
Feelings of guilt, shame, self‐criticism, low self‐worth, hopelessness Feeling isolated, lack of connection and support from friends Perceived personal stigma: own beliefs and values		Alleviate guilt, shame, stigma, low self‐worth Empathy and relating Offering sympathy Offering connection, support, hope, optimism	Lack of sympathy and/or empathy Lack of relating, authenticity or overfamiliarity in response Potentially triggering distressing emotions associated with, or invalidation of, abuse
**Fears and uncertainties around consequences of help‐seeking if perceiving outcomes to be worse**	**Own positive experiences of help‐seeking may alleviate some fears and uncertainties**	**Addressing fears and uncertainties around consequences of help‐seeking with understanding and positive shared experiences**	**Ignoring fear of consequences in giving advice**
Making situation/experiences worse for self			Offering negative responses and emotions that may exacerbate fears of help‐seekers
Uncertainty around the process, being taken into care and loss of family			
Making situation worse for family/others			
**Avoidant coping or maladaptive safety strategies inhibit help‐seeking**	**Motivations to reach out for help** a	**Empower and motivate through development of psychological competencies to counteract avoidance strategies and encourage help‐seeking**	**Discourage motivation to seek help through sharing of negative experiences**
**Knowledge, means and understanding of how to access help and support**			
**Relationships and lack of trust in confidentiality through process of disclosure**	**Feeling connected to others and availability of trusted adult support**		

*Note*: Bolded text represents key themes; unbolded text represents sub‐themes.

^a^ Evidence for an effect of peer support from thematic analysis through change in help‐seeker responses.

**Table 3:** Proposed mechanism of peer support to alleviate help‐seeker needs on the online message boards

Peer support offers	Interaction	Help‐seeker needs
Knowledge, understanding, compassionate responding and nonjudgemental shared experience from peers	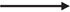	A. Support process of validation and acceptance of EM
Peers encourage recognition and expression of emotions/symptoms through compassionate responding	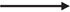	B. Develop emotional competencies and regulation
Well‐informed knowledge and positive shared experiences of *outcomes* offered by peers	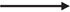	C. Alleviate fears of consequences for current, future situation, self and others
Empathetic connection to build relationships with peers in the Childline community through interactions	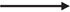	D. Counteract loneliness
Well‐informed knowledge and positive shared experience of *help‐seeking* offered by peers	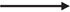	E. Enhance knowledge and trust in sources of help
Peers enhance self‐efficacy (control) and self‐esteem through social connection alleviating stigmas	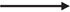	F. Motivate and empower to engage with others

#### 2.5.3 Conversation analysis

The conversation analysis aimed to determine what features of online message board chat or ‘conversation’ are most important to enable CYP to feel supported and/or facilitate an ongoing help‐seeking journey. The embedded open questions for YCoR probed the broad areas identified from existing literature (Box 1).^25^, ^29^, ^30^, ^31^, ^32^ Themes were generated with these different areas in mind. All threads were included to investigate elements that were helpful or unhelpful. Unlike thematic analysis, interactions and conversational features can be both specific to a message and/or relate to a sequence of interactions within threads and so were difficult to evidence to discrete text references. Thus, YCoR reflections were coded by the adult researcher as the ‘interpretative data’ for this analysis, tracked to corresponding threads in NVivo.

#### 2.5.4 Reiterative and reflexive processes to finalize themes from both analyses

Draft schema from both thematic and conversational analyses were extracted from NVivo to Excel to share with YCoR in the final workshop. An exercise was devised to co‐produce final themes from both analyses and explore the utility of the help‐seeking framework. This design allowed for reflective practices between the YCoR and adult researchers (V. B. and C. G.) and aimed to establish trustworthy themes and subthemes and to capture the breadth of experiences and YCoR interpretations. YCoR utilized their experience and knowledge from the initial phenomenological interpretative analyses; described in detail in Bennett et al.^18^ Themes and recommendations were finalized after a discussion session facilitated by YCoR with senior staff members of the NSPCC.

## 3 FINDINGS

This summary of findings will describe characteristics of peer interactions, evidence of change, identification of mediators of change from thematic and conversation analyses and the application of the help‐seeking framework.

### 3.1 Characteristics of roles and peer–peer interactions

Five threads involved two help‐seekers, and the rest included one help‐seeker. Threads took place over a total duration of 1–103 days between 2016 and 2019 (the majority of posts were between 2018 and 2019; the range was extended for one sample/YCoR to select neglected posts). Initial help‐seeker posts received responses after 2 h to 22 days (*n* = 18); two posts received no peer supporter responses but one of these received a host (moderator) response. Nine help‐seekers only posted their initial message. As expected, longer threads had a greater number of unique pairs of peers interacting. First, help‐seeker posts varied between 13 and 376 words. In total, 10 message threads displayed a conversation with between 4 and 14 posts involving between 1 and 5 peer supporters. Full details of interactions are shown in Supporting Information: Data Table 2 and ‘Thread numbers’ correspond to those provided in Bennett et al.^18^ for detailed contextual background.

### 3.2 Defining and evidencing change

Throughout the qualitative analyses, evidence of change was explored for help‐seekers. Change was detected through the engagement and/or interaction of members as follows.

1. Individual help‐seekers. A noticeable change in their state in 9/10 threads (composition, emotion, sentiment), or expressed intention to seek help or accept advice in sequential message responses in 4/10 threads. Many supporters and responding help‐seekers expressed gratitude to peers for listening and acknowledging the compassion received. Others returned to the board to update on progress (e.g., perspective taking following peer advice helped one help‐seeker resolve their differences with a parent).
2. Members who started out as help‐seeker and changed role. YCoR arrived with a problem to share or to seek advice but also offered support/became peer supporters during the message thread or across different threads (*n* = 3). For example, some of the primary help‐seekers responded to other help‐seekers later in the thread or on another thread encouraging them not to self‐harm and offering support (e.g., thread 1). They also provided reciprocal messages expressing their support for other help‐seekers; such as posting that (they; as an individual) or (we; as a community) are there for them, asking them to (let them know how they got on) and to ‘inbox them’ suggestive of a motivation to continue connection (note: there was no private message function).
3. A peer supporter who developed more compassionate peer responding skills. This peer supporter wrote an abrupt message on the first thread (thread 12) and received no response. They then posted a nonempathic response on another thread (thread 9) and were politely ‘called out’ by a peer who offered a more empathetic response to the help seeker. Following this interaction, the first peer supporter mirrored the approach to improve the compassionate qualities of their support and elicited a noticeable change/positive interaction with another help‐seeker to interact in a conversation later in this thread.

### 3.3 Mediators of change for help seekers identified from reflexive thematic analysis

Through the reiterative and reflexive process, mediating factors associated with change for help‐seekers. These were either explicitly stated or inferred (by the research team) as negative factors (barriers) that may prevent change in help‐seeking or positive mediating factors that may facilitate help‐seeking. From help‐seeker posts, six key themes were proposed to describe barriers that prevent CYP from progressing their ‘help‐seeking’ (Table 2; first column). The theme ‘Poor emotional competence’ was multifaceted with subthemes detailing a range of individual psychosocial factors affecting help‐seekers' understanding and expression of their emotions. This was also the most frequently referenced theme across help‐seeker posts for both barriers and facilitators (other columns). Within this theme, the subthemes most commonly referenced were ‘feelings of shame, self‐criticism, low self‐worth, and hopelessness’ alongside ‘expressed guilt’ and ‘feeling isolated’ (Supporting Information: Data Table 3).

A range of potential facilitators from help‐seekers who appeared more advanced in their help‐seeking characteristics and/or further along the process are presented in Table 2 (second column). Facilitators to help‐seeking inferred from peer supporter threads (9/13 threads associated with positive changes) are shown in Table 2 (third column). A breadth of compassionate responses included: acknowledging their own suffering from shared abusive experiences, revealing their vulnerabilities and emotions and extending this to offer and accept compassion from others. Importantly, some peers used their own experiences alongside compassionate responses to deter and offer advice and emotional support to others expressing suicidal ideation and self‐harm behaviours (relevant to the subthemes of emotional competence).

Some potential factors in peer supporter posts that YCoR suggested may exacerbate barriers are shown in Table 2; fourth column. Sharing bad experiences could also be seen to exacerbate difficulties. For example, in one thread, a peer supporter provided a negative experience of reporting abuse (i.e., associated with a bad experience of foster care) potentially compounding fears around seeking help. This was directly rebutted as unhelpful by another peer supporter, with a similar but more positive experience. They offered a more hopeful response. This also elicited a positive change in the first supporter's approach.

Table 3 summarizes how help‐seeker needs (aligned with the above themes) could be moderated by different aspects of peer support from the findings of the reflexive thematic analysis.

### 3.4 Mediators contributing to moderation of peer support from conversation analysis

Key themes and subthemes for conversational elements and qualities that may facilitate, or present barriers to, help‐seeking and the moderating effects of peer support are presented in Table 4. Examples of YCoRs explanations coded by the adult researcher supporting each of the following described themes are provided in Table 5.

**Table 4:** Conversational themes describing mediating factors associated with help‐seeking, and possible moderating effects of peer support

Conversation	Barriers to help‐seeking	Facilitators to help‐seeking
Composition of posts, thread, flow, coherence	Abrupt or impolite statements that do not facilitate conversation	More detailed (yet concise) information may facilitate better advice giving
	Conflicting advice from different PS in conversation	PS supporting each other may build confidence in advice and coherence
	Conversation may be stopped if HS feel it served purpose of getting advice	
	Lack of questions to promote further connection and interaction	Questions promote willingness to engage further, add clarity (context) and extend chat
	Reply function may confuse or inhibit some conversations	Reply function can add clarity to conversation and advice giving (and show listening and relating)
		Sharing experience helps expand conversation and increase involvement
Relationship building and rapport	Lack of information does not facilitate building trust and rapport	
	Lack of shared experience or compassion offered by PS	Courtesy, rapport, 'listening' and empathy encourage HS—compassionate responding
	Too impersonal, not authentic or not relating to the HS situation may prevent conversation	Informality of conversation or chat
Sequence organization and interactions	Lack of interaction or brief replies from HS or PS	
	More than one HS or PS in chat	More than one PS or HS in chat may offer more support
		Same people responding will help develop trust
	Not requesting a response explicitly	
	Unequal contributions may affect quality of interaction and impact of peer support	Good balance of conversation and content between individuals may help feel more supported
Temporality of responses: rapidity, duration, spacing	Delays in responding affect quality of conversation	Early and rapid responses are important for this population to feel validated, supported, part of community
	Many immediate response with advice could be overwhelming	
	Rapid but rushed replies may make them feel unimportant	Effect of timing of replies on feeling supported may be dependent on state of HS and quality of response
	Slow responses may affect feeling supported and motivation to seek help	
	Spaced out or unevenly spaced responses disrupt conversation and may affect checking boards	Close proximity (in time) of replies aids flow of conversation
		Longer conversations show change in help‐seeker

Abbreviaions: HS, help‐seeker; PS, peer‐supporter.

**Table 5:** Examples from young co‐researcher responses that support key conversational elements and qualities

Conversational attributes	Younger co‐researcher responses
Composition of posts, threads, flow, coherence	
Use of questioning in threads	Peter (16 years): ‘If the PS asks the HS a question it shows they are interested and they want to keep the conversation going because they care about the person getting better’.
	‘I think when PSs say stuff along the lines of “here for you” that is the best way for them to encourage longer interaction or conversation. It seems like a barrier for a lot of HSs is fear that they will take up too much of other people's time, so saying stuff like that is important’.
	Clara (15 years) and Sophie (17 years): ‘Lots of detail about situation—sharing emotions—(when he tells them about how his mum has “all the teachers fooled”, and how the situation is still bad, he gets quick responses’.
	Juniper (15 years): ‘Having long responses in general offers more content and advice to reply to’.
Consistency of responses and supporting each other—coherence	Asher (16 years): ‘A safe place is very important when they come onto the board … not feeling judged’.
	Peter (16 years): ‘This gives them [peer supporter] a degree of confidence in what advice they are offering … it's good for the peer supporter’.
	Heather (16 years) and Asher (16 years): ‘The more replies enforcing the same idea, the more likely that OP [original poster] is going to think they should follow that advice (could be good or bad)’.
Disrupting flow	Sophie (17 years): ‘Blunt responses/lack of emotional support. Also signing off with finality, e.g., “good luck you're stronger than him” does not encourage HS to respond’.
	Peter (16 years): ‘For example, a peer supporter started replying and talking about their own problems and it was not clear whether following replies were responding to the first problem or the second problem’.
	Juniper (15 years): ‘It can interrupt the flow of conversation slightly, as responses can become muddled and it may be confusing who the HS is replying to as they don't directly address the PSs’.
	Peter (17 years) and Simeon (14 years): ‘In some cases it can really complicate things, as people sometimes posted about their own problems in the reply function which confuses the thread, and then there can be multiple conversations going on in the same thread talking about different things which is not ideal’.
Relationship building and rapport	
Formality of conversation	Asher (16 years): ‘…important to how the conversation was flowing and also how supported the person who was posting felt. Sometimes there were posts were people were using really abbreviated terms and Latin and stuff and I don't feel that was great, not that they didn't care but didn't put as much thought into the response as they could have. Whereas if you had someone who was too formal and instructive it was overwhelming … getting it somewhere in the middle is quite important’.
	‘Colloquial language removes the background stress of structuring your point perfectly, so it encourages responses’.
	Cassia (16 years): ‘Also, when they were going more formal to not be too patronising’.
Developing rapport with compassionate responding skills	Peter (16 years): ‘Validation can come in different forms … the peer supporter was saying all the right things but the tone was coming across as very blunt and uninterested’. Clara (15 years) and Sophie (17 years): ‘Sharing emotions—when he tells them about how his mum has “all the teachers fooled”, and how the situation is still bad, he gets quick responses’. Asher (15 years): ‘Establishing a personal connection (by using names or anecdotes) is important for trust. If the HS trusts the responders, they are more likely to interact again and share more about how they feel. Using quotes from the HS's post may make them feel listened to’.
Identifying with others and mutual sharing of experience	Juniper (15 years) and Cassia (17 years):
	‘PS shared their own experience, showing that the help given is not always as drastic as the previous reply described—as they share their experience it could show the help‐seeker that they can get through it and it is possible for things to get better … [peer supporter 1] reply encouraged [peer supporter 2] to also share their experience. This might make HS more confident in telling someone about their situation. Sharing personal experiences seems to encourage a longer conversation because someone usually responds with support and then their experience so it ends up a bit like a chain’. ‘Sharing experiences can cause knock‐on effects and help others (both PSs and HSs) to also share’.
Sequence organization and interactions	
Developing trust and familiarity to feel part of a community	Peter (16 years): ‘Whilst it all is anonymous it is vital to feel comfortable responding without really knowing who they are’.
	Cassia (16 years): ‘Also builds up a friendship which is important if you are feeling lonely. If you are being emotionally abused you are probably quite lonely. If you are on the message boards you probably don't have anyone you feel comfortable talking to in real life. Good to have a back‐up friendship going on line’.
Good balance and consistency in conversation between fewer individuals	Asher (15 years): ‘It is easier to get a conversation going if there are only two people. Better to get a conversation going if not tonnes of people with opinions jumping in the way of the first one if that works’. … ‘a sequence of the same people responding instead of lots of different people (and maybe fewer rather than more would) is best because they'll become comfortable with the same people, and be speaking to a smaller audience, which will both help them be more open’.
More than one person in the interactions	Peter (16 years): ‘A big group of people supporting you could be a facilitator because it shows that a lot of people care and you can have a diverse range of advice which would be helpful because people have different experiences … On the other hand it could make the help‐seeker nervous because they may feel like they have a large audience to talk about their problem to which may be a barrier’. Asher (15 years): ‘Personally, I believe it adds to the conversation more than it disrupts it, mainly because threads between two people are usually quite short and to‐the‐point. Also, different people bring in different ideas and different anecdotal experiences, so the HS wouldn't get bored of the conversation and might keep on interacting. As long as the interjector doesn't contradict what another PS said, I think the more people on a thread the better’.
Temporality of interactions—rapidity, duration, spacing	
Early and rapid responses	Peter (16 years): ‘They are not sure of abuse. Early response is therefore vital to get through first stage of anxiety’.
	Cassia (16 years): ‘Important that someone is there straight away. If longer time period they may feel more lonely and that help is further away’.
	Heather (16 years): ‘Quick replies will create a sense of community and make the HS feel validated’.
	Asher (15 years): ‘Having quick responses may facilitate help‐seeking as the HS received immediate suggestions’.
	Simeon (14 years) and Peter (17 years): ‘The responses all came promptly, there wasn't really any delay and the conversation flowed well. This probably helped the HS feel more comfortable, which was helpful because it allowed them to reveal important context to the PS. If people are given prompt replies it allows them to feel like their concerns are valued and their problem is not just them being melodramatic (many posts had issues with confidence saying things like “to me it just feels like I'm being pathetic”). A good flow (meaning fast responses) is essential because a re‐occurring theme in responses is the knocking down of misconceptions that the HS might have about their problem, such as doubting its seriousness, thinking they haven't got options, and/or maybe thinking they can handle it themselves. We think that setting these misconceptions (barriers) straight quickly is quite important as the HS's confidence moving forwards may in some part depend on that prompt replies can be important, as they provide facilitators that are needed ASAP, such as where to seek help and what the best immediate course of action is’.
	Caitlin (17 years): ‘The comments happened shortly after the post, this may have made the HS feel listened too and like they are not alone’.
Effect of timing of replies depends on state of mind and quality of response	Sophie (17 years): ‘Although the HS may be supported by the immediacy of some responses, they were not that empathetic, and she is not really asking specifically for help, but more wanting someone to listen, so this may have had an impact on the likelihood that the HS felt supported … However quick responses that don't provide what the HS needs eg emotional support, may be detrimental to their use of the threads and therefore their likelihood of using the advice provided to get help’.

Abbreviaions: HS, help‐seeker; PS, peer‐supporter.

#### 3.4.1 Composition of posts and thread, flow, coherence

Asking questions during interactions was considered to be important to promote further engagement and extend the chat. Including more information and longer conversations were suggested by YCoR to enable peers to offer more tailored advice and options. YCoR suggested that some help‐seekers conveyed the sense of ‘being a burden’, that is, taking up time from the peer supporter. Questions from the peer supporter may help alleviate this perception indicating genuine interest in their problem.

Peers supporting each other when giving advice on the boards was suggested to improve the coherence and flow of the thread; reassuring and enabling members to feel confident and safe to offer advice in the supportive community. However, there could be negative as well as positive effects. Abrupt or impolite statements were found to have a negative effect in cutting off the conversation, disrupting flow and possibly turning help‐seekers away.

Some technical features such as the reply function could add clarity or confuse some conversations when used incorrectly.

#### 3.4.2 Relationship building and rapport

Courtesy, rapport, listening and empathy—demonstrated by compassionate responding and individualized peer responses—are important in delivering effective peer support. YCoR noted that this appears to help CYP to work towards validation of their abuse while on the boards and reduce feelings of shame. Identifying with others who convey similar concerns and experiences was also considered to be important and reflect the value of shared experience from the thematic analysis.

#### 3.4.3 Sequence organization and interactions; turn taking, who talks to who and when

A logical sequence organization with interactions between the same people (i.e., unique help‐seeker–peer‐supporter interactions) was considered to help develop trust and familiarity, along with an equal contribution of conversation between individuals. More responders in a thread may serve to alleviate loneliness and build community. However, it may be unhelpful if conflicting advice was offered by different supporters.

#### 3.4.4 Temporality of responses—Rapidity, duration and spacing

The rapidity, duration and spacing of responses were variable across the 20 threads. Timings for initial peer responses ranged from 1 h to 22 days, and threads showed regular and irregular interactions between young people over periods ranging from the same day to up to 30 days. From the analysis of the ‘conversations’, early and rapid responses were important to better support those who come to the boards questioning whether they are experiencing abuse and possibly engage those who have experienced neglect. Better support in this sense was described by YCoR in terms of help‐seekers feeling more immediately connected and ‘valued’ in the community, less alone with their problems, validated as well as expressing an intention to seek help earlier. Delays in responses or erratic spacing/timing of supporter posts were thought to have a deleterious effect on the perceived ‘support’ in terms of validation and adversely affected the quality, flow and coherence of the conversations. Additionally, the effect of timing of replies on feeling supported may also be dependent on the state of mind of the help‐seeker, particularly the quality of the response and whether it addressed the needs of the help‐seeker. For example, rushed replies lacking empathy may deter them from further engagement and help‐seeking.

### 3.5 Relational mechanisms of peer support—Connection, compassion and community

The conversational approach highlighted impactful contributions from CYP connecting with peers with different roles on the message board. Mapping common themes from thematic (experiential/content; Table 3) and conversational (interactional; Table 4) analyses identified the following attributes and qualities of supportive conversations between peers:

(1) early and continued, nonjudgemental validation of problems;
(2) compassionate response from peers;
(3) feeling connected with peers and being part of a ‘safe’ community;
(4) supportive sharing of experience and knowledge.

It was proposed that connection, compassion and community are potential relational mechanisms that may moderate change through peer support during help‐seeking.

### 3.6 Application of the help‐seeking process

**Figure 3:**
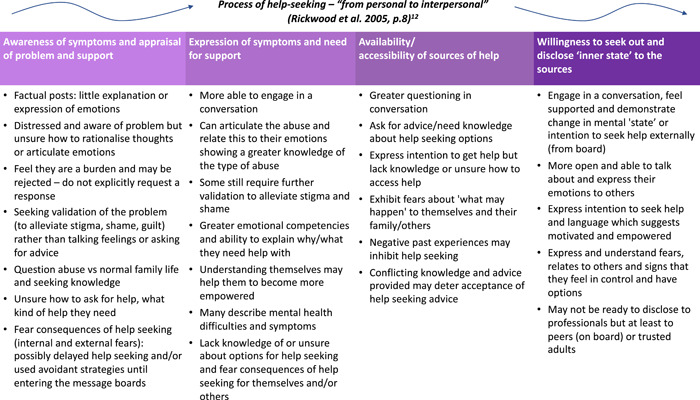
Description of characteristics discussed and inferred from posts of individuals experiencing emotional abuse and neglect on the Childline message boards and how these may relate to different stages in the help‐seeking process

Descriptions of key characteristics inferred from posts by YCoR and the adult researchers, and how these could relate to different ‘Stages’ in the help‐seeking process defined by Rickwood et al.^11^ are shown in Figure 3. The majority of help‐seekers were initially placed by YCoR at Stage 1 of the model simply by engaging with the Childline message board, and some appeared to arrive with greater psychological (emotional and motivational) capabilities at Stage 2. Many help‐seekers were considered to lack the capabilities to describe their problems and disclose their emotions to others as would be required later in the process defined by the model. The YCoR and adult researchers agreed that it was often difficult to place help‐seekers at a specific Stage; they may sit between ‘Stages’ in the process or may move back and forth as the thread progressed. Furthermore, those who had previously received or were receiving support were assumed to be in Stage four but some of these help‐seekers did not display the expected psychological characteristics to have progressed through earlier Stages of the process/model. Three examples are described in Table 6.

**Table 6:** Examples that illustrate the nonlinear help‐seeking process: YP already receiving support access the message boards

Thread no.	Description of thread
4	The help‐seeker has experienced a long history of abuse and a parent has been reported to social services. They are in contact with social services, motivated and keen to be interviewed to progress support but distressed waiting. This would suggest they have/are ready to connect with support. However, their post is very factual and doesn't specifically focus on their own internal state or describe emotions of themselves or others. They focus on changing their external environment. They are less fearful of the consequences than staying in the current environment so the off‐line motivation for help‐seeking may be more to evade the toxic environment and seek safety than being willing and capable of sharing their emotional state.
5	The help‐seeker has experienced a long history of abuse and discloses a range of mental health and emotional symptoms. They have been removed from the abusive situation and are waiting for a CAMHS appointment. However, their post also suggests that they are trying to alleviate emotional distress, seek further validation and resolve their lack of social connection through engaging on the boards.
13	The help‐seeker has experienced severe neglect and is receiving professional support (psychotherapy) which may suggest they are in the final stage of the model. However, this may not reflect their emotional competence. If the model is representative, it would be assumed they are engaging with support and have a capability and willingness to reveal their inner state. The post indicates they are still seeking validation (or perhaps invalidation) of the neglect, are in significant distress and do not disclose details of their own emotional experience. They do not respond to any peer support message.

## 4 DISCUSSION

This co‐produced study developed a novel methodological approach to better understand help‐seeking for CYP experiencing emotional abuse and neglect from online peer–peer conversations. Using this methodology, the full research team defined potential mediators (barriers and facilitators) and functions of peer support and hypothesized peer‐support mechanisms associated with change. Critical contextual and psychological evidence from the interpretative phenomenological co‐analysis is reported in detail in Bennett et al.^18^ and informed the co‐produced thematic and conversation analyses reported here; this embedded YCoRs lived experiences throughout the study.^18^, ^34^

### 4.1 Evidence of change through interactions

This co‐produced triangulated methodological approach responded to some of the challenges in researching ‘real‐world’ data to characterize change resulting from interactions, look in depth at the content meaning at a moment in time and explore the impact of conversational elements over time. The evidence of positive perceived change in state and/or intention of the individual help‐seeker to seek or accept advice following 9/10 compassionate peer conversations illustrates the positive impact that these message boards can have for CYP and further justified the methodological approach.

### 4.2 Mediating factors (barriers and facilitators) to help‐seeking and moderation by peer support

Mediating factors from message content (reflexive thematic analysis) and interactional factors (conversation analysis) were critical to providing supportive conversations in this online community. Help‐seekers need to provide enough information for a peer supporter to respond and give appropriate information and advice. Peer supporters need to provide compassionate conversational responses that facilitate the emotional competence of the poster. The impacts of multiple interactions in threads were noticed across two of the conversational themes (Table 5; ‘Composition of posts, threads, flow, coherence’ and ‘Sequence organization and interactions’). While multiple supporters may offer help‐seekers with alternative choices and greater autonomy, YCoR also commented that some help seekers may need consistent, or complementary advice from peer supporters to enhance the reliability and help‐seeker confidence in the advice being offered. Thus, multiple interactions may bring about different outcomes depending on individual help‐seekers needs and contexts.

A lack of understanding and inability to validate the abuse, or its seriousness, appeared to be a critical barrier to starting and progressing on the journey of help‐seeking.^18^ The findings support early responsiveness and validation of a help‐seeker's abusive experiences as one of the most important functions of online peer support. Through interpersonal exchanges, peer supporters facilitated the process of knowledge seeking, understanding and moving towards acceptance of emotional abuse and neglect; some shared experiences and others did not.

Sharing of experience requires a delicate balance between what may be supportive and enabling for the help‐seeker, and what may become a barrier. The nature of the experience shared and conversational delivery was found to matter; nonjudgemental, compassionate delivery conveying an understanding and relational empathy for the help‐seeker's experience was associated with better outcomes (positive changes in perceived state and/or help‐seeking intention as outlined above). Thus, when delivered in the right way, such responding may also serve to reduce perceived social stigma and negative social evaluation or comparison. Other research supports the role of telling ‘their stories’ and sharing experiences around mental health in empowering individuals, reducing perceived social stigma by removing some of the ‘direct’ fear of social judgement and improving well‐being.^39^ In addition to personal beliefs and stigmas, perceived social stigma is a significant predictor of intention to seek help in adolescence.^40^ These were evident across themes for help‐seeker and peer supporter posts in this study.

Peer supporters without experience of abuse were also able to play an important role in the validation process when responses were delivered compassionately since responses were positively acknowledged and accepted by help‐seekers in threads associated with positive change, even when experiences were not shared. These supporters highlighted unacceptable adult behaviour, offered sympathy and supported comments of others without judgement in conversations that were associated with a positive change. Such positive effects have been observed in a school setting, where ‘intergroup contact’ between those with and without mental health problems reduced stigma and benefited mental health literacy, emotional well‐being and help‐seeking attitudes.^41^ In this study, misunderstanding, unsupportive or insensitive peer responses that seemingly lacked sympathy, or motivation to empathize with the help‐seeker's experience or distress, may have adversely affected this process by invalidating their abuse or inducing negative social comparison.

These results imply that CYP need to feel safe, motivated and able to articulate their problem and symptoms (or emotions) to another person or develop this through interaction. The emotional competence of help‐seekers,^18^ and the capabilities of peer supporters to respond emotionally and supportively were considered critical in enabling or inhibiting help‐seeking journeys. Some CYP appeared to develop a greater expression of emotions through the course of conversations. However, emotional competence or a negative emotional state has also been reported to affect an individual's perceived personal stigma^40^; which in this context may relate to feeling safe or willing to articulate their emotions to others in messages. Most CYP who reported experiences of neglect in their posts used less rich emotional language, content and few or no questions in their posts, thus offering less of a ‘hook’ to engage a peer supporter in an enduring and helpful conversation (see also Bennett et al.^18^). Being unable to realize their emotional needs, and hence know what or how to ask for help, presents a significant barrier to help‐seeking for these CYP; this may also relate to the fact that many individuals often report noticing difficulties later in life.^24^, ^42^, ^43^

Competent peer supporters who elicited responses from help‐seekers used compassionate responding skills offering sympathy, empathy and relating to encouraging ‘talk’. This may have served to soothe expressed guilt and perceived social stigma that may give rise to negative emotional states through perceived personal stigma, shame and low self‐worth; noted in subthemes.^40^, ^44^ These compassionate interactions appeared to help the individual recognize some of their emotions, validate their feelings in relation to the abuse and describe some of their mental health symptoms.

### 4.3 Applying theoretical frameworks

This short study incorporated a theoretical process of help‐seeking. While Rickwood et al.'s^11^ help‐seeking process was helpful to focus the research on the inter‐ to intrapersonal process, it was challenging to apply to the online context. Although certain psychological characteristics inferred from message posts aligned to specific Stages, it was difficult to consistently relate help‐seekers and mediating factors to specific Stages or to ascertain the change in help‐seeking. The findings generally agree with the ‘needs’ outlined in the model but highlight the added complexity for CYP experiencing emotional abuse and/or neglect. Furthermore, the potential interaction between online and offline environments may affect validation and fears around seeking help away from the safety of the online community. Other researchers report that interventions may be unsuccessful if they ignore neurocognitive adaptations to abuse^45^, ^46^; often to cope with the unsafe environments.^47^ It is proposed this may affect their perceived emotional maturity (and competence) arriving at the message boards. Thus, the framework as it stands may not sufficiently account for the complexities and challenges faced by this population; during interactions or moving between different environments. The process is more dynamic—less linear—than this process suggests. These lines of evidence render the framework less useful for designing and assessing online help‐seeking interventions.

### 4.4 Relational mechanisms of online peer support—Connection, compassion, community

Considering the moderating effect of peer support in ‘helpful conversations’, three possible relational mechanisms were consistently identified across thematic and conversational analyses to support help‐seeking for these CYP.

#### 4.4.1 Connection to alleviate loneliness and facilitate sharing

Help‐seekers were motivated to actively engage with other CYP but often appeared fearful of the consequences of seeking help for themselves or others. The anonymity of a message board potentially offers safeness to connect and explore offering CYP control over when and how they interact.^18^, ^20^ It was assumed that all CYP actively posting in that moment were seeking or acknowledging connection was required to continue their journey. Connection was highlighted by YCoR as important for members: to alleviate loneliness, develop relationships and trust, facilitate sharing of experiences, develop emotional competence and/or help them feel listened to. Temporal and technical features of the online connections, notably rapidity and duration, were also found to be important.

#### 4.4.2 Compassionate motivations in interactions

Compassionate features of posts where peer supporters used their own compassionate skills—sympathy, empathy, validation and sometimes sharing of experience without judgement—to soothe negative emotions and distress (‘Emotional competence’ subtheme of peer supporter posts in thematic analysis) and ‘Relationship building and rapport’ in turn‐taking (theme in conversation analysis) were found to be associated with positive change; advancing the help‐seeking process from personal to interactional. CYP were also willing and able to learn compassionate responding skills from each other to improve their perspective‐taking and the quality of their empathic responding. Compassion relates to the care‐attachment system; specifically the developed capability and motivation to be sensitive and responsive to the suffering of oneself and others.^44^, ^48^ Grounded in evolutionary and biopsychosocial mechanisms, compassion‐focused approaches are rapidly becoming integral to therapeutic approaches to alleviate symptoms associated with trauma, self‐criticism and shame.^49^ Such engagement observed on the message boards is akin to two of the three flows of compassion: offering and accepting compassion to/from others.^48^ One study has reported lower self‐compassion associated with youths experiencing child maltreatment.^50^ In the current study, many CYP expressed gratitude for the responses they received, acceptance of support and advice and wrote of their intention to seek further help. This latter expressed intention suggests some individuals may become motivated to develop self‐compassion (third flow of compassion) following interactions.

A number of studies on adolescents suggest that self‐compassion is inversely related to internalizing symptoms, can improve resilience and be protective against psychological distress, relieve social anxiety and improve recovery from trauma.^51^, ^52^, ^53^ However, a study involving adolescents has suggested that self‐worth is an antecedent to self‐compassion and not vice versa.^54^ From the thematic analysis (and interpretative phenomenological analysis reported in Bennett et al.^18^ low self‐worth was a characteristic disclosed and/or inferred for many CYP in this study (subtheme of ‘Poor emotional competence’). Accordingly, evidence of compassionate skills and actions (as mentioned above) throughout turn‐taking was associated with positive perceived changes in help‐seeker responding (including inferred changes in ‘psychological states’) in such threads (both thematic and conversation analyses). Thus, compassionate responding through supportive connections to others may serve to enhance the help‐seeker's self‐worth enabling their own motivation for self‐compassion through mutual exchange. Evidence from a study of online peer support training suggests that being trained to offer peer support can enhance compassion for others, and also realize self‐care and well‐being within the peer supporter.^55^ YCoR themselves noted ‘supporting the supporters’ on the message boards as equally important, since many peer supporters are/were help‐seekers themselves with similar experiences. Future research could investigate the mechanisms and flows of compassion in peer support in online environments. This may be important in considering more structured roles and/or evidence‐based training for peer supporters in the context of emotional maltreatment.^18^

#### 4.4.3 ‘Safeness’ of the online community

The compassionate qualities of member interactions are integral in developing and maintaining the ‘safeness’ of the online community. YCoR felt the ‘publicly open’ and inclusive community was important to offer wider experiences, and choices and perhaps show that more people care. The importance of community was also found in a study of YP seeking help for suicide.^21^ Confidentiality and anonymity also contribute to the safeness of this online ‘holding’ community to enable some of these YP to explore and develop interrelational capabilities and skills through supportive interactions. However, YCoR highlighted a major challenge is achieving a sense of community without overburdening members, particularly peer supporters, or inducing further distress or feeling ‘loss’ when members leave the board unexpectedly. Effects of ‘anonymous’ relationships on CYP and attachment may be particularly relevant to explore for this population; there is the potential to exacerbate attachment trauma through later separations.^56^ Interestingly, some young help‐seekers (and peer supporters) post once to ask a question, or tell their story but do not engage fully in an interaction.

For more passive members, the publicly open community created on the boards may serve a purpose beyond those who actively engage to gain knowledge from peers' conversations. Outside of the social transaction (an interaction or conversation), it is possible that the message boards may function as a source of information to enhance CYPs knowledge and literacy around abuse in a ‘safer’, more passive way rather than via a more involved (formal) social transaction; as suggested by Pretorius et al.^20^ From the present study, it is unknown what drives the behaviours of those who post once or offer brief messages of support but do not become actively involved in the conversations; whether ‘learning from the boards’ is helpful and sufficient for those who are more self‐reliant or too fearful to directly engage. Thus, what this online environment offers CYP in comparison with ‘closed’ online forums and message boards (and any related help‐seeking interactions offline) requires further study. These recommendations were proposed by YCoR and researchers to warrant further exploration with young people and service users with relevant lived experience.^34^, ^57^

### 4.5 Limitations and future directions

Challenges and future directions relevant to data sampling from ‘real‐world’ data sources and participatory involvement are reported in the companion papers.^18^, ^34^ A key methodological limitation of ‘real‐world’ analysis is determining effectors of change and outcomes since it is not possible to know whether CYP contacted other services after engaging with peers. Although the change described in this paper may serve as a surrogate outcome, only so much can be inferred from this small study of written posts and threads. Co‐produced, creative methodological solutions using big data approaches (i.e., artificial intelligence and machine learning) may negate some questions over reliability if performed with ethical and safeguarding oversight. This may also facilitate the study of a larger sample size.

Insights from YCoR highlighted some potential responses from peers that may exacerbate barriers and/or negative feelings; these were considered more difficult to infer reliably with the current methodology but is also worth further follow‐up with diverse CYP and different caregiving experiences. Individual CYP will be likely to experience different challenges depending on their context, emotional competence, neurodiversity and developmental stage; some of these factors were difficult to ascertain in this study and should be considered in future research.

Finally, although the role of moderation was beyond the scope of this short project, YCoR did question if this may affect the etiquette of the board which often seemed very polite and formal. They suggested this ‘monitoring’ could affect the language and behaviours of CYP using the boards. Whether these were positive or negative effects and the contribution this made to the community, was not discussed or investigated in this project. This might be a meaningful addition to further explorations.

## 5 CONCLUSIONS

This co‐produced naturalistic study of online help‐seeking for CYP experiencing emotional maltreatment describes new methodological approaches to explore these ‘real‐world’ publicly available data sources involving YCoRs. With regard to the function of online informal support in the journey of these CYP, the most obvious is trying to help them make sense of the very complex and difficult state of affairs they find themselves in through interaction. Connection, compassion and community, which in many ways are interrelated, are potential mechanisms that could be the focus of further co‐produced research to upscale and advance this methodological approach and our understanding in this under‐researched area of help‐seeking.

## AUTHOR CONTRIBUTIONS

Vanessa Bennett was the lead researcher at the research Institution throughout the project, developed the first draft of the manuscript and coordinated the involvement of young people. Chloe Gill was involved in conceptualizing the project, contributed to and co‐facilitated all involvement sessions, developed content and reviewed all stages of manuscript development. Pam Miller was involved in conceptualizing the project, contributed to and co‐facilitated some involvement sessions and reviewed all stages of manuscript development. Peter Lewis is a young co‐researcher involved in all stages of the project, including data analysis, and in developing the outline, and reviewing various stages throughout manuscript development. The NeurOX YPAG Young Co‐researchers contributed significant involvement in data analysis and were involved in the development of the outline for the manuscript. Catherine Hamilton‐Giachritsis provided expertise in the discipline of clinical and forensic psychology to support the first author in structuring the manuscripts, particularly interpreting results and discussion; providing feedback on several draft stages. Iris Lavi provided supervision and expertise on the reporting of qualitative methodology and in emotional development and regulation to shape the interpretation of the findings for the discussion; providing feedback on several draft stages.

## CONFLICT OF INTEREST

C. G. and P. M. are employed by the NSPCC. The remaining authors declare no conflict of interest.

## ETHICS STATEMENT

The study was approved by the NSPCC Research Ethics Committee (Ref:R‐20‐189, 2020) with reciprocal review by the Secretariat of the University of Oxford Medical Sciences Interdivisional Research Ethics Committee (Ref:R62044/RE001). The study utilizes open‐access data. Service users are informed that their data may be used for evaluation and research purposes.

## Supporting information

Supporting information.

Supporting information.

## Data Availability

The data that support the findings of this study are available from the corresponding author for peer review. Some data are not publicly available due to privacy or ethical restrictions. Supplementary data is provided with the manuscript.
