# Supplementary material for: Developing a novel co‐produced methodology to understand ‘real‐world’ help‐seeking in online peer–peer communities by young people experiencing emotional abuse and neglect
Source: Health Expect. 2022 Oct 10;25(6):3124–42. doi: 10.1111/hex.13621 (PMC9700183; doi:10.1111/hex.13621)
Supplement: Supplementary file 1 — Supporting information. [file HEX-25--s002.docx]

**Supplementary Data**

**Supplementary Table 1. Terminology and definitions of emotional abuse and psychological neglect provided and discussed with YCoR**

| NSPCC definitions of abuse provided to YCoR as preparatory information. |
| --- |
| Additional information explained and discussed with YCoR from the research literature. |
| Most hidden form of abuse that results from the relationship between an adult/ parent and child |
| - Sometimes called emotional maltreatment (EMT) – some differences in definitions |
| Potentially harmful interactions which can be associated with severe impairments of a child’s emotional and developmental health, involving: |
| - omission (emotional neglect) and commission (emotional abuse) acts  e.g. spurning (rejection and dismissal of feelings and worth), terrorising, isolating, exploiting, and denying children their right to express emotions and be comforted |
| - repeated patterns of behaviour that expresses to children that they are worthless, unwanted, unloved, or only of value in meeting another’s needs, which leads to lasting damage to their well-being and development |
| Some research has shown that what children report as abuse differs to the official scales used by authorities – youth voice is rarely considered |
| Suggested that EMT differs to *“inadequate [parenting] behaviours”* by: |
| - how often, and how long it continues for |
| - how severe (??) |
| - potential harm to the child or youth |
| Research suggests “low levels” ie. one or two occurrence's per year can have a negative impact on youth |

Hart and Glaser (2011); Watters and Wojciak (2020); NSPCC web site: <https://www.nspcc.org.uk/what-is-child-abuse/>

**Supplementary Table 2. Summary of interactions, conversations and posts displaying evidence for a change in state and/or intention to seek help.**

| **Thread** | **Context of abuse (initiating help-seeker)** | **Total no posts** | **No. of HS** | **Total HS posts** | **No. of PS** | **PS replies** | **No. of interactions** | **Type (and number) of interactions**^†^ | **Conversations**^‡^ | **Evidence from conversations of:** | |
| --- | --- | --- | --- | --- | --- | --- | --- | --- | --- | --- | --- |
|  |  |  |  |  |  |  |  |  |  | **Perceived change in state of HS** | **HS accepting advice/intention to get help** |
| 1 | Domestic abuse (witness extreme violence), Psychological safety and security, acceptance and self esteem, hostility | 14 | 1 | 6 | 5 | 8 | 16 | 5 pairs: HS1 + PS1 (2) HS1 + PS2 (6) HS1 + PS3 (4) HS1 + PS4 (2) HS1 + PS5 (2) | 5 | Yes | No |
| 2 | Emotional abuse and neglect – acceptance and self esteem, negativity/hostility, threat to autonomy, ridiculing, threat to safety and security - risk of physical abuse/not feeling safe | 11 | 1 | 5 | 5 | 6 | 10 | 5 pairs:  HS1 + PS1 (1)  HS1 + PS2 (2)  HS1 + PS3 (2)  HS1 + PS4 (2)  HS1 + PS5 (3) | 4 | Yes | No |
| 3 | Emotional abuse: acceptance and self esteem, age-appropriate autonomy and restriction, ridiculing comments | 7 | 2 | 4 | 3 | 3 | 6 | 3 pairs: HS1 + PS1 (2) HS1 + PS2 (2) HS1 + PS3/HS2 (2) | 3 | Yes | Yes |
| 4 | Physical and emotional abuse: lack of psychological and physical safety, social services contacted | 7 | 1 | 4 | 3 | 3 | 3 | 3 pairs: HS1 + PS1 (3) HS1 + PS2 (3) HS1 + PS3 (3) | 3 | No | No |
| 5 | Domestic, physical and verbal – threat to psychological safety and security, acceptance and self esteem, manipulation and control by parent | 6 | 2 | 5 | 1 | 1 | 4 | 2 pairs: HS1 + PS1 (2) HS1 + HS2 (2) | 2 | Yes | No |
| 6 | Emotionally abusive/narcissistic father/hostile environment – domestic abuse (verbal) and manipulation | 5 | 1 | 3 | 2 | 2 | 4 | 2 pairs: HS1 + PS1 (2) HS1 + HS2 (2) | 2 | Yes | No |
| 7 | Doesn’t specify type or details of the abuse but has complex PTSD possibly different forms or repeated; bullying associated with bisexuality | 4 | 1 | 2 | 2 | 2 | 5 | 2 pairs: HS1 +PS1 (3) HS2 + PS2 (2) | 2 | Yes | No |
| 8 | Emotional abuse and neglect – acceptance and self esteem, negativity/hostility/manipulation of emotions, ignoring and ridiculing emotions, inhibit autonomy and socialising | 12 | 2 | 6 | 2 | 6 | 9 | 2 pairs: HS1 +PS1 (7)  HS1 + HS2 (1) HS2 + PS2 (1) | 1 | Yes | Yes |
| 9 | (HS1) Abusive parents but form not disclosed  (HS2) Seeking help for an emotionally abused friend.^¶^ | 11 | 2 | 3 | 5 | 8 | 5 | 2 Pairs: HS1 + PS3 (1) PS3 + PS4 (1) PS3 + HS2 (3) | 1 | Yes | Yes |
| 10 | Physical abuse and ignored/neglect Unsafe environment | 5 | 1 | 2 | 2 | 3 | 4 | 2 pairs: HS1 + PS1 (3) HS1 + PS2 (1) | 1 | Yes | Yes |
| 11 | Emotional abuse and neglect - acceptance and self esteem, negativity/hostility/ manipulation of emotions, psychological safety and possible physical abuse or restraint | 5 | 2 | 2 | 3 | 3 | 4 | 4 pairs: HS1 + PS1 (1) HS1 + PS2 (1) HS1 + PS3 (1) HS2 + PS2 (1) | 0 | - | - |
| 12 | Abuse of a friend – form not disclosed | 3 | 1 | 1 | 2 | 2 | 2 | 2 pairs: HS1 + PS1 (1) HS1 + HS2 (1) | 0 | - | - |
| 13 | Severe neglect – exposed to parental alcohol and substance abuse Neglected needs – caring role for siblings Physical abuse from parent’s partner and  domestic abuse | 2 | 1 | 1 | 1 | 1 | 1 | 1 pair:  HS1 + PS1 (1) | 0 | - | - |
| 14 | Emotional abuse and neglect, threatened physical abuse, witnessed domestic abuse (physical and verbal/ emotional/controlling) towards parent | 5 | 1 | 1 | 4 | 4 | 4 | 4 pairs: HS1 + PS1 (1) HS1 + PS2 (1) HS1 + PS3 (1) HS1 + PS2 (1) | 0 | - | - |
| 15 | Emotional abuse – no further details | 3 | 1 | 1 | 2 | 2 | 2 | 2 pairs: HS1 + PS1 (1) HS1 + PS2 (1) | 0 | - | - |
| 16 | Witnessed emotional and physical domestic abuse/violence and potentially entrapment | 4  (1 Mod) | 1 | 1 | 2 | 2 | 3 | 3 pairs: HS1 + PS1 (1) HS1 + Mod (1) HS1 + PS2 (1) | 0 | - | - |
| 17 | Neglect, physical and emotional abuse from sibling and parents  Neglect – clothes, hygiene  Financial constraints  Teased/bullied - associated with neglect | 3 | 1 | 1 | 2 | 2 | 2 | 2 pairs: HS1 + PS1 (1) HS1 + PS2 (1) | 0 | - | - |
| 18 | Physical and emotional abuse from older sibling  ignored and ridiculed by parent – rejection of emotions Feeling isolated  Cultural shaming/stigmatising | 3 | 1 | 1 | 2 | 2 | 2 | 2 pairs: HS1 + PS1 (1) HS1 + PS2 (1) | 0 | - | - |
| 19 | Neglect and emotional abuse  Child protection for ACE with parent – abuse and neglect.  Exposure to risk by parent | 2  (1 Mod) | 1 | 1 | 0 | 0 | 0 | 1 Mod | 0 | - | - |
| 20 | Emotional/verbal abuse | 1 | 1 | 1 | 0 | 0 | 0 | NA | 0 | - | - |

HS = help-seeker; Mod = moderator; PS = peer support

^†^An interaction is an exchange between a unique HS-PS or PS-PS pair (i.e. at least 2 posts). This can be different PS responses to the same HS post.

^‡^A conversation is a series of interactions that includes at least one reply from the HS in the thread (at least 3 posts/2 interactions).

^¶^The context for the second help-seeker is included since this resulted in a conversation and demonstrated a change in state. No conversations were reported for second help-seekers across other threads.

Further details relating to threads 1 to 20 are provided in the corresponding thread numbers in [Author removed]. 2022.^19^

**Supplementary Table 3. Codebook for themes, subthemes and descriptions identified from thematic and conversation analyses**

**(Supply as PDF of Excel sheet)**
