# Supplementary material for: Developing a novel co‐produced methodology to understand ‘real‐world’ help‐seeking in online peer–peer communities by young people experiencing emotional abuse and neglect
Source: Health Expect. 2022 Oct 10;25(6):3124–42. doi: 10.1111/hex.13621 (PMC9700183; doi:10.1111/hex.13621)
Supplement: Supplementary file 2 — Supporting information. [file HEX-25--s001.pdf]

Supplementary Table 3. Codebook for themes, subthemes and descriptions identified from thematic and conversation analyses

THEMES and SUBTHEMES	DESCRIPTION	Threads	References
BARRIERS			
**Help seeker posts**			
Unable to understand, self-validate or accept experience of abuse and/or neglect	Related to shame and possibly guilt - guilt if they felt they could have done something to stop the abuse for them/others<br>Also fear of not being believed by others<br>Describe emotional abuse<br>Difficult to accept so look to others for validation<br>Reference to intangible nature of abuse<br>Others questioning if normal family life<br>Own beliefs - distinct from public stigma - lack ability to rationalise and form beliefs.<br>Not wanting to believe never properly nurtured - not accept feeling unloved - attention	8	11
Negative perceptions that others will have of them (perceived public stigma)	Do not feel they will be/are believed by others may affect their self-identity and self-esteem	6	6
Poor emotional competence	Emotional competence - Individual factors affecting understanding and expression of emotions		
Lack of understanding of distressing emotions associated with abuse			
Feeling isolated, lack of connection and support from friends	Isolation and also loss of trust in others/friends that they felt were close<br>Lack of connection to others/peers	7	8
Feelings of guilt	Describing feelings of guilt - feel they should have done something about abuse<br>Or explicitly stating 'feelings of guilt'<br>Though some of these latter cases may be more reflective of embarrassment or shame - belief that they are behaving ungratefully because what they see as their basic/material needs are met (not emotional needs) - they describe 'feeling bad' - possibly need to differentiate these.	4	8
Feelings of shame, self criticism, low self-worth, hopelessness	Uses self critical language and/or appeasement suggesting that they have low self worth (safety strategy to avoid being rejected)<br>Feeling to its their fault/made to feel its their fault<br>Feelings of shame - driven by embarrassment, insecurity, feeling inadequate, self doubt (could also be associated with stigma and difficulties in validating abuse)<br>This could drive self criticism/low self esteem and thus feeling unworthy/undeserving of help	10	19
Perceived personal stigma_own beliefs and values	Evidence of own beliefs towards abuse and own identity<br>Feels shame/embarrasment about some of feelings and therefore communicating the problem and their emotions to others is more difficult	4	4
Unable to describe or express emotions _ possible emotional dysregulation			
Confused or not express emotions_potential internalisation	Does not describe emotions while explaining the problem - factual posts (even describing horrific events)<br>Underlying reasons - evidence of internalisation - lack of emotional content (factual), withdrawal, self harm, depression, wanting to sort problem out on their own (various reasons - fears), possibly not explicitly asking for help. More likely to internalise and find it difficult to reach out to others and find support<br>Dissociate themselves from problem/expeirences - safety strategy<br>Not expressing anger as may be dangerous therefore internalise - safety strategy<br>Some may not have developed the ability to recognise and label their own emotions - lack of nurture (neglect)	3	3
Rejected or ignored feelings and_lack of nurture_insecure attachment	Feelings of YP are rejected and failure of parent to respond empathically to YP<br>Feeling unheard<br>May prevent from reaching out for fear of lack of response<br>Exacerbate feelings of loneliness<br>Problems with attachment here may also leave the individual with feelings associated with feeling unloved - particularly where neglect is involved (see evidence from neglect threads)	6	9
Externalising_anger	Hide vulnerability? Alternative safety strategy to internalisation - express anger and problems mis-perceived as behavioural and compliance problems	1	1

Fears and uncertainties around consequences of help-seeking if perceiving outcomes to be worse	A number of internal fears associated with possible outcomes<br>Also emotional - role of attachment and emotional stability and security<br>Related to their safety<br>Confidentiality and anonymity of boards - linked to control and choice<br>Linked to beliefs about themselves<br>Uncertainty of the process and potential outcomes associated with their family					
Making situation/experiences worse for self	Include fears about exacerbating abuse<br>Fear for own safety	6	10			
Making situation worse for family/others	This could include others involved/being abused further as a consequence of them disclosing to others	2	4			
Uncertainty around the process, being taken into care and loss of family	Lack of understanding of the process and possible fears of 'losing their family', or who they despite finding situation difficult to unbreable, and also emotional consequences associated with guilt, shame, fears etc.	3	5			
Means to access to help	This could be physical means - restricted access to outside support, entrapment, availability of phone/methods to access and contact help	3	3			
Avoidant coping or maladaptive safety strategies inhibit help-seeking	Avoidant coping & safety strategies - desire to deal with problems on their own<br>Safety strategies - avoid change as more fearful<br>Reliance on friend/s may prevent HS from finding help - Not motivating additional external help	3	4			
Relationships, trust and lack of confidentiality in disclosure	Lack of 'trust' in others and possible options for help seeking - relates to emotional competence and their Fears of consequences. (described in the Individual Factors)<br>May help drive their desire to deal with the problems on their own and avoidance strategies	3	5			
Knowledge - don't know how to get help	Lack of knowledge around what support is available, what options there are for them, how much choice they will have	4	5			
Peer advice or help not helpful, acceptable, not supportive	Evidence that they reject advice<br>Also evidence that they have no confidence in support suggested	3	5			
**Peer supporter posts**						
Lack of understanding or sensitivity to HS experience, invalidating abuse, or inducing social comparison of experience	Possibly due to lack of shared experience in some cases<br>Possibly invalidating experience of HS					
Ignoring fear of consequences in advice	Suggesting confrontational action (with abuser)	3	4			
Misreading HS post or not responding appropriately		2	2			
Potentially triggering distressing emotions associated with validation	e.g. neglect - this individual may not want it to be validated - will feel unloved<br>Or<br>Invalidating or not confidently validating abuse or neglect	3	3			
Negatively impact emotional competence						
Exacerbate distress through lack of compassionate skills and responding						
Lack of relating, authenticity or overfamiliarity in response	If the response seems overpersonal - seems to suggest they know the person, overfamiliar. Perhaps suggesting they know the person very well.	1	1			
Lack of sympathy and or empathy		5	5			
Ignoring fear of consequences in giving advice	Offering negative responses and emotions that may exacerbate fears of help-seekers<br>Negative experiences of help seeking associated with help seeking and consequences<br>This could be worries associated with fear or also of loss and pain of losing their family<br>Also lead to them blaming themselves	4	4			
Discourage motivation to seek help through sharing of negative experiences	Offering experience that may be perceived as worse	3	3			
**FACILITATORS**						
**Help seeker posts**						
Understand, self validate and begin to accept experience of abuse and, or neglect	Probably several stages here in validation and acceptance of the abuse<br>Able to describe the abuse<br>Able to connect the abuse to their own emotional states, feelings, thoughts and reactions<br>Able to free themselves from blame to accept that the abuse is not their fault or due to a ‘defect’ in their own self<br>Knowledge about problem - recognise and can articulate the problem and give evidence of symptoms that need addressing (but not all yet able to express emotions)	9	18			
Adequate emotional competence	Emotional competence - individual factors affecting understanding and expression of emotions and symptoms<br>Articulates and feels comfortable expressing symptoms and emotions to others<br>Recognises need for support/change to environment to alleviate own emotional distress (relates to Stage 2)					

Identify and talk about emotions, mental health symptoms and problems	Can identify and express emotions (around abuse) as well as self-harm, suicide and other mental health problems that are associated with stigma and shame and talk openly to others about these. This suggests they may be further along the help seeking pathway - greater capacity to engage with idea and potential benefits of support	8	21			
Understanding emotions associated with emotional manipulation	Identify the problem and describe some emotions that they have linked directly to the abuse - starting to understand some of the emotional reactions they are experiencing	3	8			
Own positive experiences of help-seeking may alleviate some fears and uncertainties	Alleviate fears and uncertainties around consequences of help seeking through positive past experiences	1	2			
Motivation to reach out for help or accept help	Shows a motivation to reach our for help<br>Experience of help seeking - normalise help seeking (appear not to be negative experience - no evidence that it is a barrier)<br>Evidence of specific requests for help<br>Possibly also motivation to change	6	7			
Feeling connected to others and availability of trusted adult support	This may reduce feelings of isolation and allow them to counteract feelings of low self worth<br>Showing compassion to others helps development of self compassion?	1	2			
Change in help seeking or accepting advice given by peer supporters	Acknowedge advice given is useful the HS has expressed an intention to change their HS behaviour	4	5			
Feeling validated, supported or change in state	Peer support helps to validate that it is not them which may help to alleviate guilt, shame, low self worth<br>Evidence from initially distressed post to expression of gratitude and connection in response(s)	9	15			
**Peer supporter posts**						
Offering knowledge and shared experience to help validate and move towards acceptance of abuse/reduce social stigma						
General validation of abuse (without relevant shared experience)		7	11			
Validating with relevant shared experience	As long as not invalidating or inducing comparison (ie. inducing feelings that abuse is not serious enough)	5	7			
Enhance emotional competencies						
Encourage talking about/expressing emotions	Acknowledging emotions<br>Help recognise and understand emotions and why they feel this way<br>Expressing their own emotions and that this is acceptable and understandable may encourage sharing of emotions	8	15			
Using own compassionate skills to soothe negative emotions and distress	Aspects and skills used to address HS concerns/barriers and encourage help seeking	4	5			
Alleviate shame, stigma and self worth	Reduce blame - not their fault<br>Positive responses to sharing story<br>Stigma - public - not comparing 'severity' of experience with others stories and experiences<br>Not separated out personal and public stigma here. Make them feel less alone by sharing similar experiences of abuse and coping mechanisms to enable them to seek help and talk about them.<br>Self worth - encouraging their need and right to get help - that they deserve to get help as much as anyone else<br>Reducing blame and shame - it's not their fault they have had this experience - it has been done to them	7	30			
Concern for distress and safety		6	7			
Empathy and relating		10	23			
Offer connection, support, hope, optimism	Offers of support and positivity - alleviate loneliness from their situation	10	24			
Sympathy	Demonstrates sympathy for situation	11	15			
Addressing fears and uncertainties around consequences of help-seeking with understanding and positive shared experiences	Understanding of situation and fears<br>Offer knowledge - situation and process or different types of help seeking and possible outcomes/consequences<br>Links to empathy and compassion for the HS situation	8	26			
Empower and motivate through development of psychological competencies to counteract avoidance strategies and encourage help-seeking						
Counteract avoidance strategy		2	2			
Encourage empowerment and self control	Counteract fears - what feels manageable to the HS<br>Potential barriers associated with low self esteem, self worth<br>Encourage problem solving_reasoning_HS choices<br>Encourage taking control - feeling of control over situation - empowered to ‘accept’ and seek help	9	16			
Knowledge and experience of help seeking		4	5			

Conversation analysis facilitators				Conversation: thread numbers	No conversation: thread numbers	
Composition of posts, thread, flow, coherence	Greetings and return greetings, content and level of information<br>Questions & answers - response/reaction as expect - coherence<br>Requests & granting of requests					
More detailed (yet concise) information may facilitate better advice giving	This could be from the HS and PS posts<br>More advice for HS to reply to in longer PS posts<br>More detail likely to be helpful to provide better advice<br>Encourages longer or more helpful conversations<br>Detail from PS explicitly describing what is likely to happen helpful to PS	6	7	2,3,6,9	11,18	
PS supporting each other may build confidence in advice and coherence	Expanding on PS and building experiences to provide more options and depth to conversation<br>PS supporting each other may encourage different experiences/perspectives and build confidence of HS to share<br>More replies from different PS reinforcing same ideas could offer confidence to HS about advice - more acceptable	4	4	9	11,14,18	
Questions promote willingness to engage further, add clarity (context) and extend chat	Questions to the HS are important to keep the conversation going and enable the PS to give emotional support and expand shared experience<br>Also clarify situation before giving advice<br>Asking questions at the end of the post helpful	4	4	3	11,13,18	
Reply function can add clarity to conversation and advice giving (and show listening and relating)	Possibly a way of highlighting points and adding empathy<br>Ensure clear which comment is being addressed -	7	11	1,6,8,9	11,14,18	
Sharing experience help expand conversation and increase involvement	Sharing experience can encourage others	3	6	6,9	11	
Relationship building and rapport	Role of sympathy and empathy - when and how offered<br>How do they show they are listening - reflecting and mirroring<br>How do they relate - shared experience or common interests or coping strategies?					
Courtesy, rapport and listening and empathy encourage HS - compassionate responding	Empathy encourages longer interactions and conversations<br>HS less likely to feel like a burden<br>Feeling that the PS has taken the time to read (and sometimes quote) and relate to the HS problem and feelings<br>Sharing emotions, establishing connection, showing sympathy for their situation, reassures the HS about sharing and establishes trust<br>Making them feel listened to	7	13	1,2,8,10	16,18,19	
Informality of conversation or chat	Colloquial language in messaging - helpful to reduce stress about getting 'wording' right, may encourage those more anxious to seek help	2	2	1	18	
Sequence organisation and interaction	How do the HS and PS interact and what effect may this have on the conversation and feeling supported or on help seeking responses: who talks to who and when (trun taking, what happens when others join in, pairs of interactions, etc)					
Good balance of conversation and content between individuals may help feel more supported	Relates to flow and interaction between messages - using information from messages	1	1	1	18	
More than one PS or HS in chat may offer more support	Offers more diverse range of views, advice, experiences, ideas<br>Creates community<br>Adds/expands conversation<br>Can change approach/help train other PS	5	7	1,8,9	11,16,18	
Same people responding will help develop trust	Sequences with same people responding build trust and confidence and open conversation	1	1	8		
Temporality of responses_ rapidity and duration	Duration, Spacing, Rapidity - what is the impact of these on conversation and feeling supported/help seeking					
Close proximity (in time) of replies aids flow of conversation	Enhances flow and opens up/expands on replies to keep conversation going	3	3	8, 10	16	
Early and rapid responses are important for this population to feel validated, supported, part of community	Fast responses that aim to reassure the HS that their problem is serious may help HS to feel more comfortable and continue conversation<br>Also many immediate responses may make them feel 'heard' in community (M16HF - no conversation though)<br>Feel listened to, less alone (shared experience also), increase likelihood of conversation - close to posting means more likely to be checking<br>Feel more cared about<br>Provide facilitators to help seeking quickly<br>Increasing confidence in engaging on boards quickly important<br>concerns valued/validated<br>Demonstrates committment to support HS	10	15	6,8,9,10	11,13,14,15,16,17	

Effect of timing of replies on feeling supported may be dependent on state of HS and quality of response	Early and rapid responses alone not sufficient if messages not empathetic or responding to needs of the HS<br>Fast responses that aim to reassure the HS that their problem is serious may help HS to feel more comfortable and continue conversation<br>Also many immediate responses may make them feel 'heard' in community (M16HF - no conversation though)	3	4	1	11,14	
Longer conversation shows change in help seeker	Reminders/reinforcement to seek help<br>Longer conversations can allow for developments in the HS situation to be discussed and accounted for in advice giving	3	3	3	14,15	
**Conversation analysis barriers**				Conversation: thread numbers	No conversation: thread numbers	
Composition of posts, thread, flow, coherence	Greetings and return greetings, content and level of information<br>Questions & answers - response/reaction as expect - coherence<br>Requests & granting of requests					
Abrupt or impolite statements that do not facilitate conversation	Greetings and endings can also affect the tone of the message. Some endings such as 'Good luck' give a sense of finality/farewell and may stop the connection/interaction<br>Very blunt responses, even if trying to support validation, if not delivered with empathy or emotional quality may not be that helpful and prevent the interaction from continuing.<br>Impoliteness/tone of message may affect usefulness of advice delivered and likelihood of response/conversation<br>Perhaps perceived as a lack of investment in the support they are offering<br>Longer messages with more content, and more advice, offer more to reply to than short responses<br>Factual responses repeating previous content that don't add to the conversation	7	9	6,9	11,13,14,17,18	
Conflicting advice from different PS in conversation	Conflicting advice from different PS may confuse help seeker or reduce confidence in help seeking and support	1	1	6		
Conversation may be stopped if HS feel it served purpose of getting advice	This can be a barrier and a facilitator to getting more help - may be individual	1	1		15	
Lack of questions to promote further connection and interaction	Without questions it is difficult to expand the conversation or add more information to the conversation. Makes it more difficult to respond. Stops a conversation developing or closes down the conversation if no further questions asked.<br>Prevents natural opening up of HS.	4	5	6	13,15,17	
Reply function may confuse or inhibit some conversations	May distract attention from the HS towards the PS experiences - risk the HS is forgotten<br>Can confuse the thread: when many different problems become posted in the same thread	6	9	3,8,9		
Relationship building and rapport	Role of sympathy and empathy - when and how offered<br>How do they show they are listening - reflecting and mirroring<br>How do they relate - shared experience or common interests or coping strategies?					
Lack of information does not facilitate building trust and rapport	Could be due to short thread or just quality of information if HS feels nothing constructive to offer may not attempt to build relationship.<br>Also provides less content on which to base a conversation, or may be misinterpreted as judgmental/confusing/intimidating and prevent building trust required to have a conversation.<br>When only brief information is given by PS who state they have experienced something similar, unless they provide sufficient information the HS may think this is normal and not as bad as it seems. This social comparison and fear of judgement may prevent them from building a relationship and continuing conversation.	3	3	1,10	11	
Lack of shared experience or compassion offered by PS	Lack of sympathy, empathy - affects the 'tone' of the message and could cause the HS to move away from the platform.<br>Some examples suggest that too much relating their own experience to others may have a negative effect if not delivered in an understanding, compassionate and empowering way. This could be without acknowledging individual circumstances or perhaps their fears of consequences, particularly if the PS has conveyed their own situation negatively.<br>If the PS convey offers a very directive advice approach that is devoid of choice, this may have a distressing effect and leave the help seeker feeling they have no control or choice over the situation and may prevent help seeking/responding.<br>Vague or short emotionless responses do not tend to get replies from HS	6	12	2,3,9	14,17	
Too impersonal, not authentic or not relating to the HS situation may prevent conversation	Poor relating whereby PS talks more generally or about themselves rather than talking directly to HS may disengage HS from conversation<br>Using overfamiliar language and trying too hard to relate may be perceived as lacking authenticity and become too personal/overpowering (e.g. a particular PS is ignored but other PS receive a reply).	2	2	7	18	

Sequence organisation and interaction	How do the HS and PS interact and what effect may this have on the conversation and feeling supported or on help seeking responses: who talks to who and when (turn taking, what happens when others join in, pairs of interactions, etc)					
Lack of interaction or brief replies from HS or PS	Thanking for support but not initiating further chat<br>Obviously general lack of replies inihibits comversation, or only single replies from PS to initial HS prevents development of conversation.	3	3	6	16,18	
More than one HS or PS in chat	May add some confusion as conversation gets broken, and may also confuse HS with regard to different options for help seeking being offered. Breaks up and discourages follow up questions being answered.<br>But could also be useful to feel more people willing to support them.<br>Sometimes it may confuse the thread but possibly not have an impact on the way the HS feels.<br>Lots of messages at once may be overwhelming for both the HS and PS.	6	6	1,3,6	13	
Not requesting a response explicitly	This may limit responses?<br>No hook to start or continue a conversation	4	5	From discussion: across threads - not identified in questions		
Unequal contributions may affect quality of interaction and impact of peer support	Unequal contributions may affect the balance, power and relationship in the conversation and may be detrimental to the flow and feel of the message boards	1	1		16	
Temporality of interactions	Duration, Spacing, Rapidity - what is the impact of these on conversation and feeling supported/help seeking					
Delays in responding affects quality of conversation	May discourage or disrupt conversation	1	1		13	
Many immediate response with advice could be overwhelming	Effects of too much advice which may adversley affect decision making	1	1		11	
Rapid but rushed replies may make them feel unimportant		1	1	9		
Slow responses may affect feeling supported and motivation to seek help		4	6	1,2	11,13	
Spaced out or unevenly spaced responses disrupt conversation and may affect checking boards	Could affect confidence in using boards<br>Unevenly spaced responses e.g. a few quick responses and then long delay than another response... separate conversations within same thread<br>Interaction of timing with other factors - sequence and composition	5	6	1,2,3,9	11	
